# Good outcomes of pregnancy in two cases of women treated with omalizumab due to the severe asthma

**DOI:** 10.1186/2045-7022-3-S1-P25

**Published:** 2013-05-03

**Authors:** Izabela Kuprys-Lipinska, Piotr Kuna

**Affiliations:** 1 Medical University of Lodz, Dept of Internal Medicine, Asthma and Allergy, Poland

## 

The prevalence of asthma during pregnancy is estimated on 4%. Uncontrolled, severe asthma is a risk for both a mother and an unborn child. Therefore, optimization of asthma treatment during pregnancy is vital in achieving good outcomes. Physicians and their patients may have some doubts about safety of anti-asthmatic medications, especially if drugs are new. Omalizumab is a humanized recombinant anti-IgE monoclonal antibody recommended for the treatment of chronic severe IgE-mediated asthma. Clinical and observational studies confirmed its effectiveness in improving asthma control, reducing severe exacerbations and improving quality of life. The safety data concerning pregnancy come from the experimental studies on animals and they show no teratogenic effect. We present case reports of two women who became pregnant during the treatment with omalizumab. Both with very severe asthma treated chronically with all available medication including systemic steroids (first – 20 mg prednisolone/day, second – 40 mg prednisolone/day). Both started their regular treatment with omalizumab in 2007 and have significant improvement (withdrawal of oral steroids or significant reduction of their dose, better asthma control) . First, 32-year old woman became pregnant in 2010 and gave birth in Oct 2010 - it was her 3rd pregnancy, and 3rd labor , second 31-year old – also became pregnant in 2010 and gave birth in Jan 2011 - it was her 5th pregnancy and 2nd labor. Both had complications during previous pregnancies and labors and decided to continue therapy with omalizumab. First woman, besides omalizumab, was treated with high doses of ICS and LABA, second - high doses of ICS, LABA and 5 mg prednisone/day. The pregnancies proceeded without asthma exacerbations and other complications. First woman delivered healthy girl (Apgar 9, weight 3200 g, length 56 cm) in 40 week of pregnancy by caesarean section due to the narrow pelvis, second - health boy (Apgar 9, weight 3800 g, length 56 cm) in 40 week by caesarean section due to the aggravating obstetrical history. In both cases treatment with omalizumab did not affect pregnancies and newborns.

